# The Significance of Superficial and Tendon Reflexes in the Lower Extremity

**Published:** 1904-02-06

**Authors:** 


### THE SIGNIFICANCE OF SUPERFICIAL AND TENDON REFLEXES IN THE LOWER EXTREMITY.

In an address delivered before the Anglo-American Medical Association in Berlin, Professor Oppenheim[1] discussed the diagnostic significance of the various reflex phenomena that are met with in the lower extremity. In the case of the knee-jerk he thinks it may be taken as true that, in the absence of any local mechanical conditions which might prevent the phenomenon, the tendon-reflex is constantly present in healthy individuals; the deficiency of this reflex is therefore a symptom of the greatest worth. Professor Oppenheim has met with apparent exceptions to this rule in two or three people in whom from early youth this reflex could not be demonstrated, but in these cases there have been present marked stigmata of degeneration. There is another class in which the patellar reflex may be absent without any concomitant signs of disease, namely, the children of syphilitic parents. In such cases there is always the possibility that other symptoms of nervous disease depending upon congenital syphilis may develop later in life. Among the purely mechanical conditions which may render the demonstration of this reflex difficult or impossible, are included obesity of such extent that it is impossible to percuss the tendon, diseases of the knee-joint characterised by swelling and deformity, marked cases of genu valgum, and a few instances where the patellar tendon is abnormally short.

The reflex obtained by percussing the tendo Achillis had up till quite recently been much neglected owing to the belief that it was quite inconstant in healthy individuals. A modification in the method of examination, namely, with the patient in a kneeling position, has shown that the reflex can be regularly excited, and the former belief in its inconstancy is no longer tenable. The reflex is always present in the healthy. The apparent exceptions to this rule are important. They include cases of sciatica, of various deformities of the foot, such as talipes valgus, and inflammatory affections of the ankle-joint, while a marked development of varicose veins appears to weaken the reflex. Even after recovery from sciatica the heel-phenomenon may remain permanently absent. Where none of these factors is present, the absence of the phenomenon is of great importance, especially in regard to the diagnosis of incipient tabes dorsalis, since recent observations have shown that the heel-jerk may disappear a long time before the patellar reflex. It is important, however, to bear in mind that alcoholism, diabetes and other affections may produce the same symptom.

Of the skin reflexes, Babinski's sign has attracted most attention. Under normal conditions when the sole of the foot is stroked, plantar flexion of the toes occurs, with dorsal flexion of the foot. Babinski was the first to recognise these two separate movements, and he further observed that in certain pathological conditions, these movements are altered so that dorsal flexion of the toes, and especially of the great toe, results from cutaneous stimulation of the sole. Babinski's sign is met with in a variety of conditions. There are reasons for believing that the phenomenon is dependent upon a lesion of the pyramidal tracts ; thus it is markedly present in spastic paraplegia, both of spinal and cerebral origin. The symptom is not due merely to hypertonicity of the muscles, because it is found in lesions which result in hypotonus or atony. That Babinski's reflex is present in the first year of life may be explained by the fact that at birth the pyramidal tracts are not completely developed. If it be assumed that Babinski's sign is due to affection of the pyramidal tracts it might be concluded that the reaction always indicates an anatomical lesion of these tracts. This is not so. There are other conditions under which the phenomenon appears, notably in epileptics and after epileptic seizures.

There is another cutaneous reflex, which was first observed by Professor Oppenheim. It is this : by lightly stroking the inner surface of the leg from above downward, or by pinching the skin in this region, in a normal individual, either no reflex movement will follow or plantar flexion of the toes will result. Under pathological conditions—that is to say in affections of the pyramidal tract—a dorsal flexion of the toes and foot is observed. The sign, being of a grosser nature than the plantar reflex, may be of value in cases where the Babinski reaction is uncertain.

[1] Medical Record, Jan. 2, 1904.

